# Size-Adjustable Microdroplets Generation Based on Microinjection

**DOI:** 10.3390/mi8030088

**Published:** 2017-03-12

**Authors:** Shibao Li, Deyin Zheng, Na Li, Xuefeng Wang, Yaowei Liu, Mingzhu Sun, Xin Zhao

**Affiliations:** 1 Institute of Robotics and Automatic Information System (IRAIS), Nankai University, No. 94 Weijin Road, Nankai District, Tianjin 300071, China; 2120150343@mail.nankai.edu.cn (S.L.); zhengdy@mail.nankai.edu.cn (D.Z.); lina@mail.nankai.edu.cn (N.L.); wangxuefeng@mail.nankai.edu.cn (X.W.); liuyaowei@mail.nankai.edu.cn (Y.L.); sunmz@nankai.edu.cn (M.S.); 2 Tianjin Key Laboratory of Intelligent Robotics (TJKLIR), Nankai University, No. 94 Weijin Road, Nankai District, Tianjin 300071, China

**Keywords:** microdroplet generation, Laplace pressure, critical injection

## Abstract

Microinjection is a promising tool for microdroplet generation, while the microinjection for microdroplets generation still remains a challenging issue due to the Laplace pressure at the micropipette opening. Here, we apply a simple and robust substrate-contacting microinjection method to microdroplet generation, presenting a size-adjustable microdroplets generation method based on a critical injection (CI) model. Firstly, the micropipette is adjusted to a preset injection pressure. Secondly, the micropipette is moved down to contact the substrate, then, the Laplace pressure in the droplet is no longer relevant and the liquid flows out in time. The liquid constantly flows out until the micropipette is lifted, ending the substrate-contacting situation, which results in the recovery of the Laplace pressure at the micropipette opening, and the liquid injection is terminated. We carry out five groups of experiments whereupon 1600 images are captured within each group and the microdroplet radius of each image is detected. Then we determine the relationship among microdroplet radius, radius at the micropipette opening, time, and pressure, and, two more experiments are conducted to verify the relationship. To verify the effectiveness of the substrate-contacting method and the relationship, we conducted two experiments with six desired microdroplet radii are set in each experiment, by adjusting the injection time with a given pressure, and adjusting the injection pressure with a given time. Then, six arrays of microdroplets are obtained in each experiment. The results of the experiments show that the standard errors of the microdroplet radii are less than 2% and the experimental errors fall in the range of ±5%. The average operating speed is 20 microdroplets/min and the minimum radius of the microdroplets is 25 μm. This method has a simple experimental setup that enables easy manipulation and lower cost.

## 1. Introduction

Microdroplet generation is a blossoming research field that presents great potential for high-throughput chemical and biological analysis, synthesis of advanced materials, digital chemical analysis, protein crystallization, and encapsulation of cells [1,2,3,4,5]. The microdroplet is a kind of ideal micro-reactor; it has been used to research microscale conditions in chemistry and life science fields. Moreover, the size of the microdroplets is unknown for some research experiments, such as chemical communication between bacteria [6]. Therefore, the size must be able to be adjusted flexibly, which increases the importance of adjusting size.

Currently, microdroplet generation frequently adopts the technology of droplet-based microfluidics or digital microfluidics, which is a subclass of microfluidic devices, wherein droplets are generated using T-junctions [7,8,9] or flow-focusing devices [10,11]. In a microfluidic flow-focusing device, the microdroplets or plugs streak under an oil layer. Various strategies are demonstrated to fabricate structures in confined spaces via fluid flow. As a unique type of structure [12], the droplets are written to Petri dish by streaking tips coupled with the microfluidic streak plate. In the microfluidic streak plate, arrayed microdroplets can be readily generated by shearing a stream of aqueous solution into a stream of oil inside microchannels. The resulting aqueous microdroplets are monodispersed and dispensed from the stream of aqueous solution at a certain frequency, then, the droplets exiting the Teflon tubing were left behind and formed a sessile-droplet array. However, once the microfluidic chips have been fabricated, the width of the channel in the microfluidic chip cannot be adjusted anymore, which limits the adjustment of the microdroplet size. To overcome the limitations in microfluidics, such as inflexibility, complexities, and expensive equipment, microinjection is regarded as a promising tool for microdroplet generation.

Microinjection for microdroplet generation still remains a challenging issue caused by Laplace pressure. The Laplace pressure of the interface at the micropipette opening cannot be removed and recovered in time, causing the uncontrollability and time-delay of the microinjection.

Herein, we apply the substrate-contacting method to handle the Laplace pressure at the micropipette’s opening and present a size-adjustable microdroplet generation method based on a critical injection (CI) model, simulating the contact printing method [13]. Firstly, the micropipette is adjusted to a preset injection pressure, which is less than CI pressure. Secondly, the micropipette is moved down to contact the substrate, which results in the collapse of the interface at the micropipette opening and the liquid flows out in time. Then, maintaining the substrate-contacting situation of the micropipette, the liquid in the micropipette flows out under pressure; therefore, the microdroplet’s radius increases gradually. Finally, the micropipette is lifted from the substrate to end the substrate-contacting condition, which results in the microdroplet dispensing from the micropipette, the interface at the micropipette opening recovers in time, and the Laplace pressure is relevant and balances the injection pressure, ceasing the flow of liquid.

We carry out five groups of experiments based on the substrate-contacting method with a micropipette where 1600 images are captured within each group and the microdroplets’ radii of each image are detected. Then we determine the relationship among the microdroplets’ radii, the radius at the micropipettes opening, time, and pressure from the experimental results. To verify the relationship, we conduct two more experiments, and the excellent quality of fit for the results verifies the universality of the relationship. The microdroplet size is adjustable according to the relationship, which is the critical step in the adjustment of the microdroplet size.

To verify the effectiveness of the relationship and the substrate-contacting method, we conduct two experiments where six desired microdroplets’ radii are set in each experiment. According to the relationship, we adjust the injection time with a given injection pressure in one of the experiments and adjust the injection pressure with a given injection time in the other experiment. Then, we obtain six arrays of microdroplets in each experiment. The standard errors of the microdroplets’ radii are less than 2% and the experimental errors between the mean and desired microdroplet radii fall in the range of ±5%. The average operating speed is 20 microdroplets/min and the minimum radius of the microdroplets is 25 μm.

## 2. Materials and Methods 

### 2.1. The Critical Injection Model

The surface-to-volume ratio of the fluid increases greatly at the micron scale and the interfacial tension plays the dominant role. Thus, Laplace pressure by oil-water interfacial tension becomes the main resistance to liquid injection. It seems that the equilibrium state is difficult to break when the micropipette is immersed in the oil without contacting the substrate. The injection pressure is increased gradually, and the liquid will be ejected when injection pressure is greater than a certain pressure, which is defined as the CI pressure. This critical certain condition, the transition from an equilibrium state to an injection state, is defined as the CI state. The injection pressure is less than the CI pressure, which ensures an equilibrium state of the micropipette, and the liquid will not be extruded from micropipette before contacting the substrate.

When the micropipette contacts the substrate, the interface at the micropipette opening collapses and the Laplace pressure in the droplet is no longer relevant as a result of the intermolecular forces between liquid molecules and the substrate molecules. Meanwhile, the equilibrium state switches to the injection state, and the liquid in the micropipette is extruded by injection pressure. Finally, the micropipette is lifted from the substrate to end the contacting situation, which results in the fast recovery of the equilibrium state and the liquid injection is terminated.

Herein, we quantify the CI pressure at the CI state using the force-balance principle. A positive output pressure is provided to balance the Laplace pressure at the micropipette opening and keeps the liquid in an equilibrium state. This positive pressure is described as the CI pressure *P_I_* in this research.

**Figure 1:**
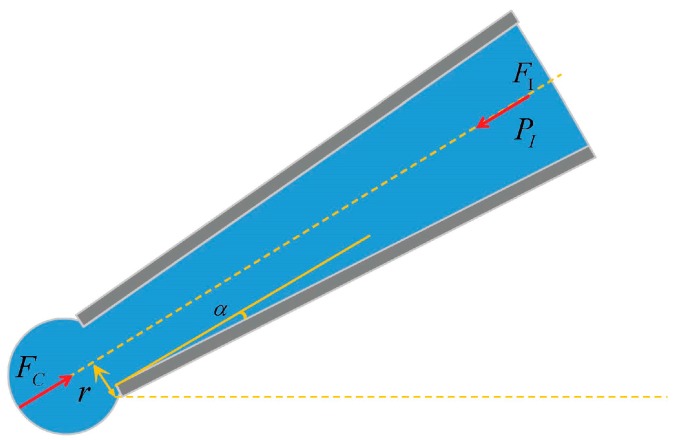
Schematic of the critical injection (CI) model.

Figure 1 illustrates the force balance of the CI state after *P_I_* has been exerted. Ignoring the friction force of the inner surface, *P_I_* is determined according to:

(1) $$P_{I}=F_{C}/\pi r^{2}$$

According to the interface mechanics, the Laplace pressure of the interface at the micropipette opening *F_C_* is estimated by:

(2) $$F_{C}=\sigma 2\cos\alpha r\pi$$

where *r* is the inner radius at the micropipette opening, α is half of the micropipette’s convergence angle, and σ is the coefficient of the water-oil interfacial tension.

Substituting Equation (2) into Equation (1), the CI pressure is:

(3) $$P_{I}=\sigma 2\cos\alpha /r$$

Since the micropipette has been lubricated sufficiently in the trypsin solution, the friction force between the liquid and micropipette is not considered. In addition, the gravity and the hydrostatic force is negligible at this scale. Moreover, to determine CI pressure *P_I_*, only the stationary equilibrium states of the liquid are considered. Furthermore, the forces generated in the dynamic process are neglected in the equilibrium state.

A group of CI pressures are quantified with different inner radii at the micropipette’s opening according to Equation (3). Subsequently, the quantitative control of the CI pressure is also achievable.

### 2.2. Substrate-Contacting Method of Microdroplet Generation

**Figure 2:**
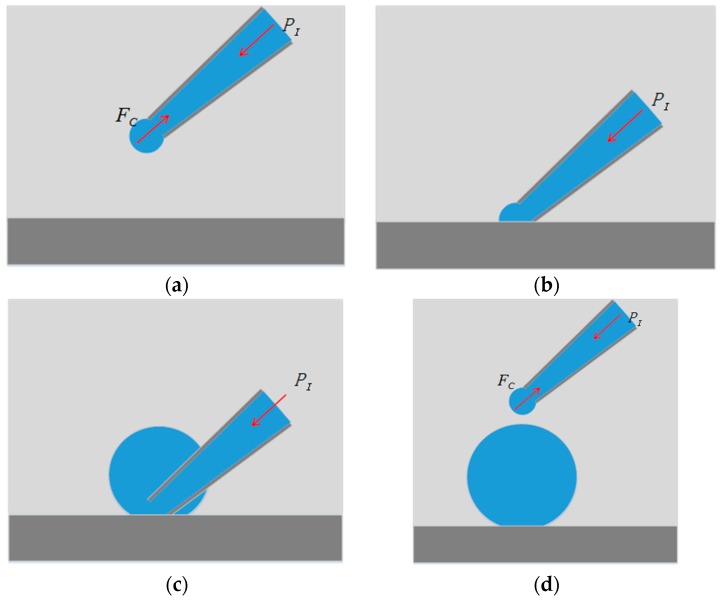
Substrate-contacting method. (**a**) The micropipette is immersed in the oil-phase liquid medium and adjusted to a certain injection pressure; (**b**) the micropipette is moved down to contact the substrate, resulting in the collapse of the interface at the micropipette opening; (**c**) the Laplace pressure in the microdroplet is no longer relevant once the liquid contacts the substrate, allowing the liquid to flow out from the micropipette; (**d**) the micropipette is lifted, causing the microdroplet to dispense from the micropipette, and the Laplace pressure reappears and balances the injection pressure; thus, the micropipette returns to the equilibrium state.

The substrate-contacting method is illustrated in Figure 2. There are four steps:

(1) The micropipette is immersed in the oil-phase liquid medium and adjusted to a preset injection pressure.
(2) The micropipette is moved down to contact the substrate, then, the interface at the micropipette opening collapses as a result of the intermolecular forces between the liquid molecules and the substrate molecules.
(3) Maintaining the substrate-contacting situation of the micropipette, the liquid in the micropipette flows out under injection pressure; therefore, the microdroplet radius increases gradually.
(4) The micropipette is lifted to end the substrate-contacting situation when the microdroplet radius reaches the desired size; thus, the microdroplet dispenses from the micropipette. The Laplace pressure of the interface balances the injection pressure, which ceases the liquid injection.

## 3. Results

### 3.1. System Setup and Materials

#### 3.1.1. System Setup

**Figure 3:**
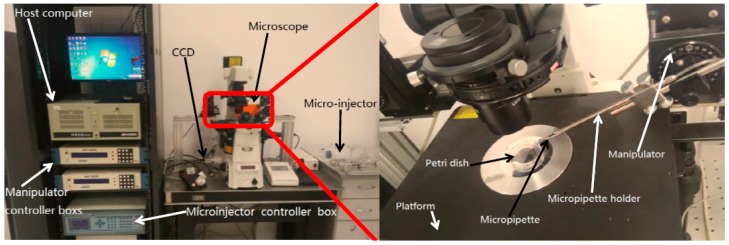
The NK-MR701 micro-operational system.

The proposed substrate-contacting method for microdroplet generation is performed by the NK-MR701 micro-operation system, which is developed by our laboratory. The system setup is shown in Figure 3. It consists of an inverted microscope (TiE, Nikon, Tokyo, Japan), a charge-coupled device (CCD, Basler AG, Ahrensburg, Germany) for microscope image-gathering at 100 frames/s, a manual *X*-*Y* stage (with a travel range of 70 mm × 50 mm and a repeatability of ±10 μm) for positioning the hybrid substrate, which is placed in a 35 mm Petri dish and covered by paraffin oil, a pair of *X*-*Y*-*Z* micromanipulators (with a travel range of 25 cm, a maximum speed of 2.9 mm/s, and a repeatability of ±0.04 μm) for positioning micropipettes, and a host computer for microscopic image processing, pressure data acquisition, and motion control of the manipulators.

A pneumatic micro-injector is developed by our laboratory, providing required pressures in the generation of microdroplets with a measurement resolution of less than 1 Pa, a pressure range of −30–150 kPa. The injector consists of a larger syringe and a smaller syringe, and the larger is taken as the buffer tank. Meanwhile, the larger and smaller volume of the tank are used to further increase the resolution and pressure range, respectively. The output pressures are adjusted through the adjusting valves equipped in each channel. Through changing the angular displacement of a stepper motor, the movement of the tank plunger is allowed for fast and coarse tuning of the pressure, while the syringe supply fine tunes the pressure. Moreover, the injection pressure is constant throughout the injecting process, utilizing the proposed pneumatic micro-injector. In microinjection experiments, the proposed micro-injector generates constant pressures and, since liquid volumes injected out are typically of the order of microliters, the displacement of the liquid does not realistically change the volume of the air trapped inside the system; thus, the pressure applied remains constant throughout the injecting process.

#### 3.1.2. Materials

The micropipettes used in the microdroplet generation experiments are made from borosilicate glass tubes with an outer diameter of 1 mm and an inner diameter of 0.6 mm. These micropipettes are first pulled by a micropipette puller (MODEL P-97 Sutter Instrument, Sutter, Novato, CA, USA). Then, they are fractured by a micro-forge (MF-900 NARISHIGE, NARISHIGE, Tokyo, Japan) to generate the required inner radius at micropipette opening. Before mounting on manipulators, these micropipettes are treated with 5% trypsin solution for half an hour to clear the inner surface of the micropipette and remove any contaminants on it. The injection liquid is deionized water, and the 35 mm Petri dish is covered by paraffin oil.

#### 3.1.3. The Hybrid Substrate

The fixation of microdroplets is the basis of microdroplet positioning. To do this, the substrate must be hydrophilic, which counteracts the random drifting of microdroplets. However, the hydrophilic substrate causes an obtuse contact angle with microdroplets. Scientific studies generally demand spherical microdroplets, that is, the contact angle must be an acute angle. 

**Figure 4:**
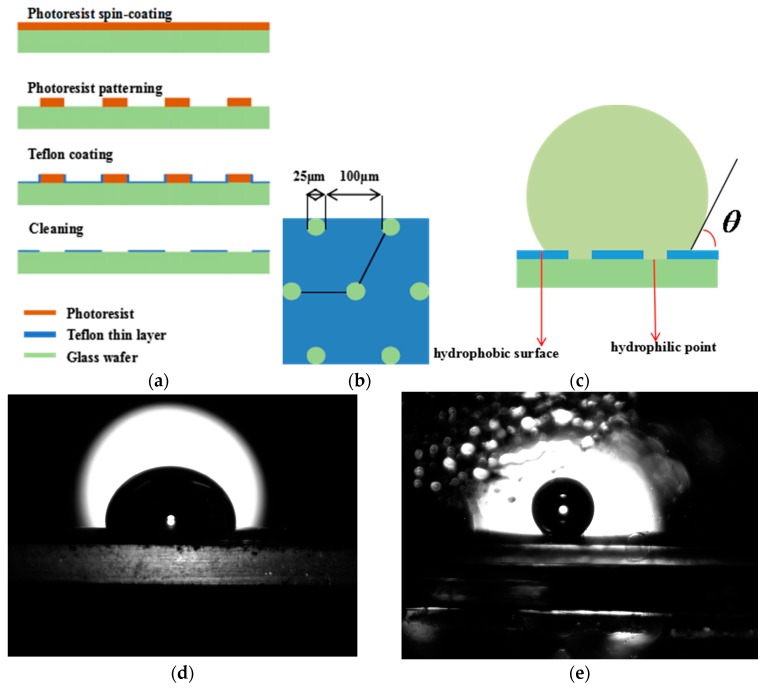
The hybrid substrate. (**a**) The detailed fabrication process flow of the substrate; (**b**) the structure of the substrate; (**c**) the microdroplets on the substrate; (**d**) the side view of the microdroplets on the substrate, in air; (**e**) the side view of the microdroplets covered by oil.

Herein, a hybrid substrate combining hydrophilicity and hydrophobicity is developed for the fixation of microdroplets. This hybrid substrate is fabricated on a bare four-inch glass wafer, on which the coated Teflon layer acts as the hydrophobic section and the exposed glass section as the hydrophilic point. Figure 4a shows the detailed fabrication process flow of the substrate.

Prior to depositing the positive photoresist, the wafer is cleaned by compressed air and a hexamethyldisilazane (HMDS) layer is deposited on the wafer to serve as an adhesion layer for the photoresist. Briefly, a 7 μm thick photoresist layer is spin-coated on the wafer with the spin-coating facility, conducted at 3000 rpm for 30 s. The wafer is then soft-baked on a hot plate at 90 °C for 90 s. Then, the coated wafer is exposed to ultraviolet (UV) light under the chrome mask, and the photoresist is developed by immersing the wafer in a 2.38% tetramethylammonium hydroxide (TMAH) solution at room temperature (RT) for 30 s. After this, the developed wafer is hard-baked on a 110 °C hot plate for 2.5 min. A 0.5 wt % Teflon solution is coated on the patterned wafer. Next, the secondary coated wafer is placed on a 150 °C hot plate for 3 min. After this step, the exposed part of the glass wafer and the side of the photoresist columns are coated in a thin Teflon layer which shows good hydrophobicity. To obtain the hydrophilic section, we need to remove the remaining photoresist from the previous step by immersing the wafer into an acetone solution and isopropanol solution each for 5 min, separately. Then the wafer is rinsed with deionized water thoroughly. Repeat this step twice until there is no remaining photoresist.

The structure of the hybrid substrate shown as Figure 4b, the diameter of the hydrophilic points is 25 μm, the space between each is 100 μm. The hydrophobic surface between the hydrophilic points cause an acute contact angle and the hydrophilic point achieves the fixation of microdroplets as shown as Figure 4c. The resulting substrate causes an obtuse contact angle with the microdroplets, as shown as Figure 4d. The microdroplets are spherical on the resulting substrate, covered by oil, as shown as Figure 4e.

### 3.2. Critical Injection Experiments

**Figure 5:**
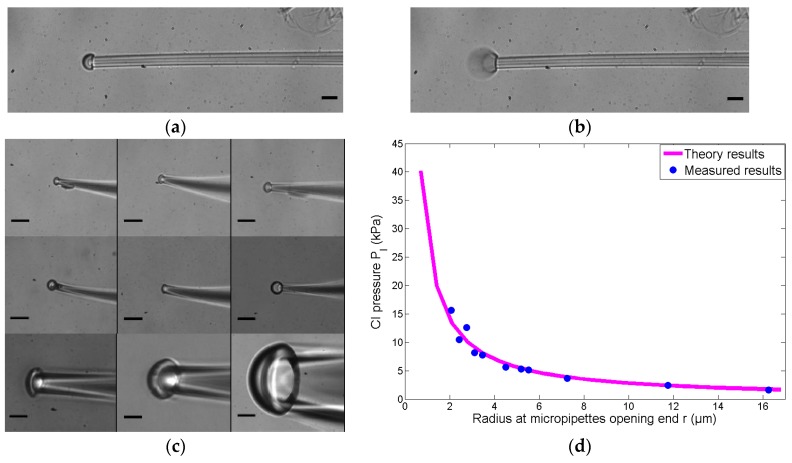
The critical injection experiments. (**a**) The state at *t_n_*_−1_, the moment before the certain moment *t_n_*_−1_, is defined as the CI state. (**b**) The liquid will be injected out at a certain moment *t_n_*. (**c**) The micropipettes in the CI state in the critical injection experiments; the scale bar is 10 μm; (**d**) the results of the CI experiments. The theoretical CI pressures (the pink curve), according to Equation (3), are compared with the measured ones (the blue points). Parameters: σ = 16.56 N/m, cosα ≈ 1, β = 30°, ρ = 998.2 kg/m^3^.

The range of the inner radius at the micropipette opening is 2 μm to 16 μm, the accurate value of the inner radius is measured through image processing algorithms in experiments. The process for each experiment is recorded as real-time images by a CCD at a frame rate of 16 frames per second. At the beginning, no positive pressure is provided to balance the Laplace pressure at the micropipette opening. Then, positive pressure is increased step by step by adjusting the stepper motor connected with the pneumatic syringe, and the liquid will be injected out at a certain moment *t_n_*, as shown as Figure 5b. The state at *t_n_*_−1_, the moment before the certain moment *t_n_*_−1_, is defined as the CI state, which is shown as Figure 5a. The pressure measured by a baroceptor at *t_n_*_−1_, the moment before the certain moment *t_n_*_−1_, is defined as the CI pressure.

A group of several micropipettes with different radii at the micropipette openings are adjusted in the CI state, as shown as Figure 5c. The CI pressure of different radii micropipette openings are shown in Table 1. Then, the CI pressure versus the different radii of the micropipette openings are calculated according to Equation (3), which is shown in Figure 5d (the pink curve).

Experimental results in Figure 5d show that the measured values are almost identical to the theory results, and the average value of error is 7%. Therefore, in the following experiments, we obtain the CI pressure according to Equation (3) instead of measuring the CI pressure of the micropipettes one by one.

### 3.3. Size Adjustable Microdroplets Generation Based on the Substrate-Contacting Method

#### 3.3.1. Microdroplet Generation Based on the Substrate-Contacting Method

We conducted five groups of experiments for microdroplet generation based on the substrate-contacting method. The radius at the micropipette opening was 2.2 μm. We chose five injection pressures with equal intervals, which are 12.67 kPa, 11.18 kPa, 9.70 kPa, 8.21 kPa, and 6.73 kPa, respectively. They are all less than the CI pressure, according to Equation (3). Firstly, the micropipette is positioned and adjusted to a certain injection pressure. Secondly, the micropipette is moved down to contact the substrate, which causes the liquid to flow out, and maintaining the substrate-contacting situation for 100 s. Finally, the micropipette is lifted from the substrate, ending the liquid injection. 

**Figure 6:**
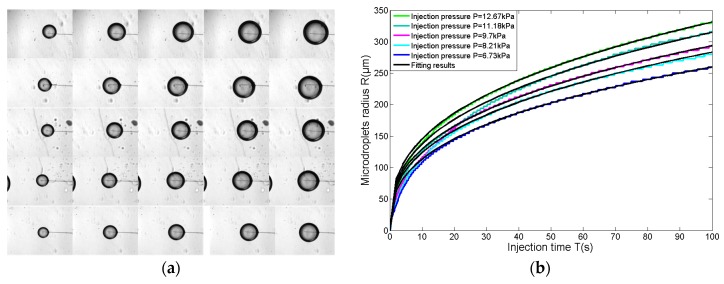
Microdroplet generation based on the substrate-contacting method, where the radius at the micropipette opening is 2.2 μm. (**a**) The images of microdroplets at 20 s, 40 s, 60 s, 80 s, and 100 s, and the five rows of images correspond to the five groups of experiments, respectively; (**b**) microdroplets’ radii of five groups of experiments within 100 s. They are fitted with the equations in Table 2 (the black curve), with *r*^2^ > 0.98.

The process for each experiment is recorded as real-time images by CCD with 16 fps; thus, the injection process of 100 s is captured in the 1600 images. The images for microdroplets at 20 s, 40 s, 60 s, 80 s, and 100 s are shown in Figure 6a. The 1600 images for each experiment are processed with a circle detection algorithm, then the microdroplets’ radii are detected and shown in Figure 6b. 

Experimental results in Figure 6b show that microdroplet’s radii follow a timescale-free increase. From this, a power-law increase of the microdroplet radius is desired under the fixed pressure. Hence, the relationship between the microdroplets’ radii and the injection time is fitted with Equation (4):

(4) $$R_{r}=a_{r}\times T^{b_{r}}$$

where *b* is an exponent, *a* is the amplitude correction coefficient, *R* is the radius of the microdroplet, *T* is the injection time, and *r* is the radius at the micropipette opening. The fitting results for five groups of experiments are shown in Table 2, the quality of fit to the data from the five groups are all excellent, with correlation coefficient *r*^2^ > 0.98.

**Figure 7:**
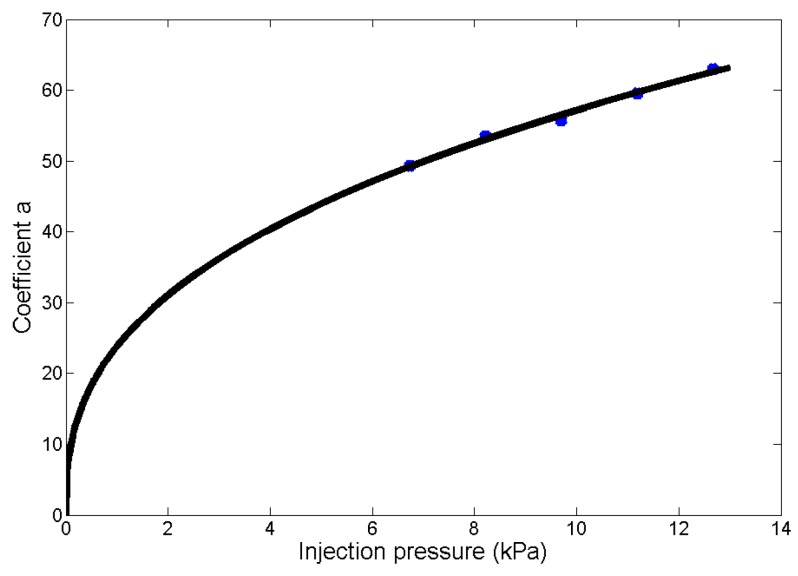
The coefficient *a* follows the power law versus injection pressure (the blue points), which is fitted with Equation (5) (the black curve), with a goodness of fit of 0.99.

Furthermore, the experimental data in Figure 7 shows that the coefficient *a* (the blue points) closely follow the power law versus injection pressure *P*, which fits (the black curve in Figure 7) with Equation (5), with a goodness of fit being 0.99. Substituting Equation (5) into Equation (4), the relationship among the microdroplets’ radii, injection pressure, and injection time is determined with Equation (6).

(5) $$a_{r=2.2}=23.83P_{r=2.2}^{0.38}$$

(6) $$R_{r=2.2}=23.83P_{r=2.2}^{0.38}T_{r=2.2}^{0.36}$$

To verify the relationship, we conduct two more experiments for microdroplet generation, with the radii of the micropipette of 3.5 μm and 6.9 μm, respectively. 

**Figure 8:**
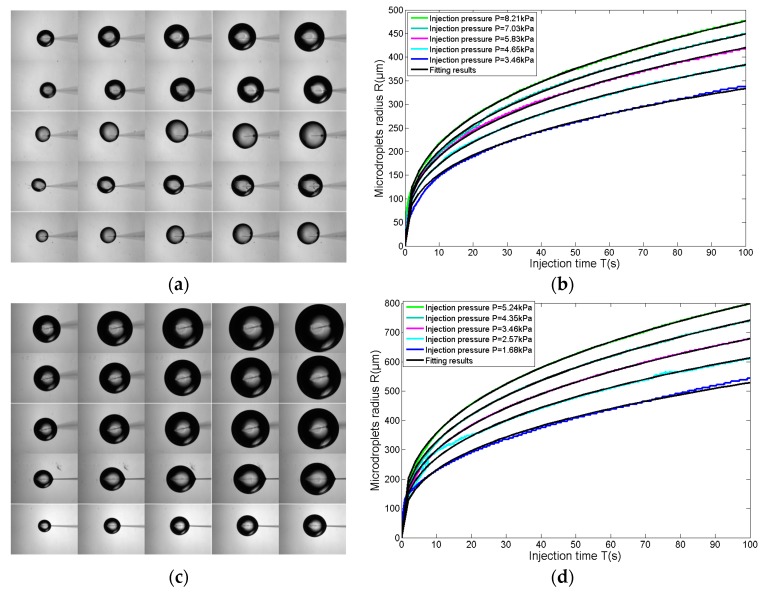
Microdroplet generation based on the substrate-contacting method. (**a**) The images of microdroplets at 20 s, 40 s, 60 s, 80 s, and 100 s, and the five rows of images corresponding to the five groups of experiments, respectively. The radius at the micropipette opening is 3.5 μm; (**b**) microdroplet radii of five groups of experiments within 100 s. They are fitted with the equations in Table 3 (the black curve), with *r*^2^ > 0.99; (**c**) the images of microdroplets at 20 s, 40 s, 60 s, 80 s, and 100 s, and the five rows of images corresponding to the five groups of experiments, respectively. The radius at the micropipette opening is 6.9μm; (**d**) microdroplet radii of five groups of experiments within 100 s. They are fitted with the equations in Table 4 (the black curve), with *r*^2^ > 0.99.

In the second experiment, the radius of micropipette is 3.5 μm and the five groups of experiments correspond to five fixed pressures, which are 8.21 kPa, 7.03 kPa, 5.83 kPa, 4.65 kPa, and 3.46 kPa, respectively. The process for each experiment is recorded as real-time images by CCD at 16 fps. Thus, the injection process of 100 s is captured in 1600 images. The images for microdroplets at 20 s, 40 s, 60 s, 80 s, and 100 s are shown in Figure 8a. The 1600 images for each experiment are processed by a circle detection algorithm. Then, the microdroplets’ radii are detected and shown in Figure 8b. Experimental results in Figure 8b show that microdroplet radii follow a timescale-free increase. From this, a power law increase of microdroplet radii is desired under the fixed pressure. Hence, the relationship between the microdroplet radius and the injection time is fitted with Equation (4), and the fitting exponents for time and pressure must be consistent with the first experiment, as *T*^0.36^ and *P*^0.38^.

**Figure 9:**
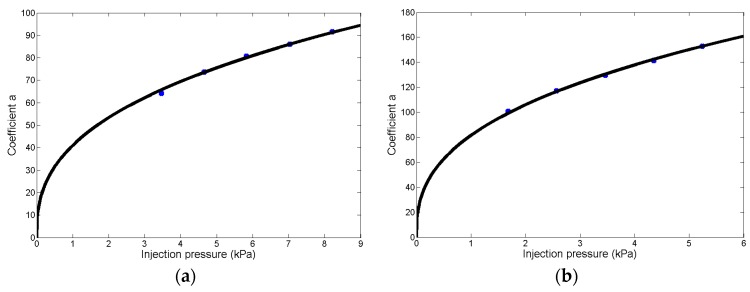
The coefficient *a*. (**a**) The coefficient *a* follows a power law versus injection pressure (the blue points), which is fitted with Equation (7) (the black curve), with a goodness of fit of 0.99. The radius of the micropipette opening is 3.5 μm; (**b**) the coefficient *a* follows a power law versus injection pressure (the blue points), which is fitted with Equation (9) (the black curve), with a goodness of fit of 0.99. The radius of the micropipette opening is 6.9 μm.

The fitting results for five groups of experiments are shown in Table 3, and the quality of the fit to the data from the five groups are all excellent, with *r*^2^ > 0.99. Furthermore, the experimental data in Figure 9a shows that coefficient *a* (the blue points) closely follows a power law versus injection pressure *P*. Thus, when fitted (the black curve in Figure 9a) by Equation (7), the goodness of fit is 0.99. Substituting Equation (7) into Equation (4), the relationship among the microdroplet radius, injection pressure, and injection time is determined with Equation (8).

In the third experiment, the radius of the micropipette is 6.9 μm and the five groups of experiments correspond to five fixed pressures, which are 5.24 kPa, 4.35 kPa, 3.46 kPa, 2.57 kPa, and 1.68 kPa, respectively. The process for each experiment is recorded as real-time images by CCD at 16 fps. Thus, the injection process of 100 s is captured in 1600 images. The images for microdroplets at 20 s, 40 s, 60 s, 80 s, and 100 s are shown as Figure 8c. The 1600 images for each experiment are processed by a circle detection algorithm, then, the microdroplet radii are detected and shown in Figure 8d. Experimental results in Figure 8d show that microdroplet radii follow a timescale-free increase. From this, a power law increase of microdroplet radii is desired under the fixed pressure. Hence, the relationship between the microdroplets’ radii and injection time is fitted with Equation (4), and the fitting exponents for time and pressure must be consistent with the first experiment, as *T*^0.36^ and *P*^0.38^.

The fitting results for five groups of experiments are shown in Table 4, the quality of the fit to the data from the five groups are all excellent, with *r*^2^ > 0.99. Furthermore, the experimental data in Figure 9b shows that coefficient *a* (the blue points) closely follows a power law versus injection pressure *P*. Thus, when fitted (the black curve in Figure 9b) by Equation (9), the goodness of fit is 0.99. Substituting Equation (9) into Equation (4), the relationship among the microdroplets’ radii, injection pressure, and injection time are determined with Equation (10).

(7) $$a_{r=3.5}=40.98P_{r=0.35}^{0.38}$$

(8) $$R_{r=3.5}=40.98P_{r=3.5}^{0.38}T_{r=3.5}^{0.36}$$

(9) $$a_{r=6.9}=81.4P_{r=6.9}^{0.38}$$

(10) $$R_{r=6.9}=81.4P_{r=6.9}^{0.38}T_{r=6.9}^{0.36}$$

The excellent quality of fit for the two experiments’ results verify the universality of the relationship. Furthermore, the amplitude coefficients in Equations (6), (8), and (10) follow a linear law versus radius at micropipette opening *r*, with a goodness of fit of 0.99. Thus, the relationship among *R*, *T*, *P*, and *r* is determined with Equation (11).

**Figure 10:**
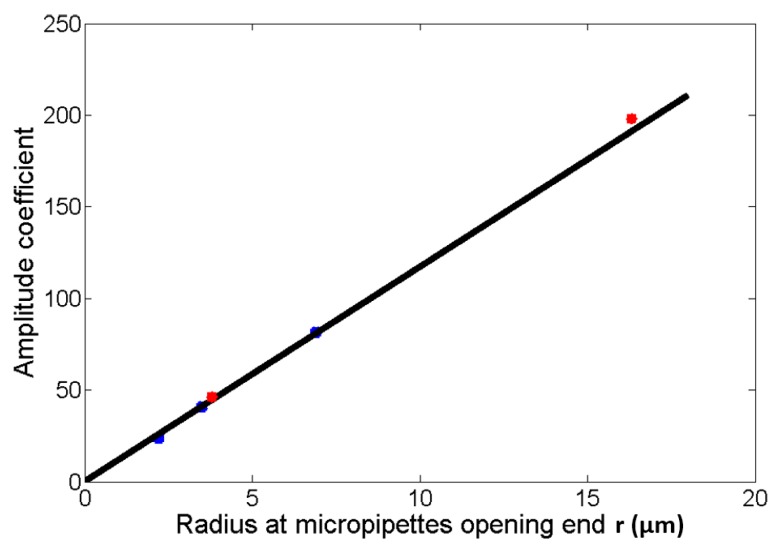
The amplitude coefficient versus radius at the micropipette opening *r*. The blue points are the amplitude coefficient in Equations (6), (8), and (10). The black line is the fitting result. The red points are the amplitude coefficient versus *r* being 3.8 μm and 16.3 μm, respectively.

Here, we perform another two experiments with the radius of micropipette opening being 3.8 μm and 16.3 μm, respectively. The microdroplet radii are generated with a given injection time and injection pressure. Thus, we can determine the amplitude coefficient versus *r*, as shown in Figure 10. The amplitude coefficient versus 3.8 μm micropipette and the 16.3 μm micropipette are almost identical with the theoretical results, according to Equation (11), and fall in the range of ±4%. Therefore, in the following experiments, we obtain the relationship among *R*, *T*, *P*, and *r* according to Equation (11).

(11) $$R=11.71rP^{0.38}T^{0.36}$$

Consequently, Equation (11) is rearranged to compute the injection time to obtain a desired size of microdroplet at any given injection pressure, or to compute the injection pressure to obtain a desired size of microdroplets at any given injection time, which are the critical steps of size-adjustable microdroplets generation.

#### 3.3.2. Size-Adjustable Microdroplet Generation Based on the Substrate-Contacting Method

To verify the relationship among microdroplet radius, time, and pressure, we conduct two experiments of size-adjustable microdroplet generation based on the substrate-contacting method. We adjusted the injection time with a given pressure to obtain six desired microdroplet radii in the first experiment, and the injection pressure is adjusted with a given time to obtain six desired microdroplet radii in the second experiment. 

**Figure 11:**
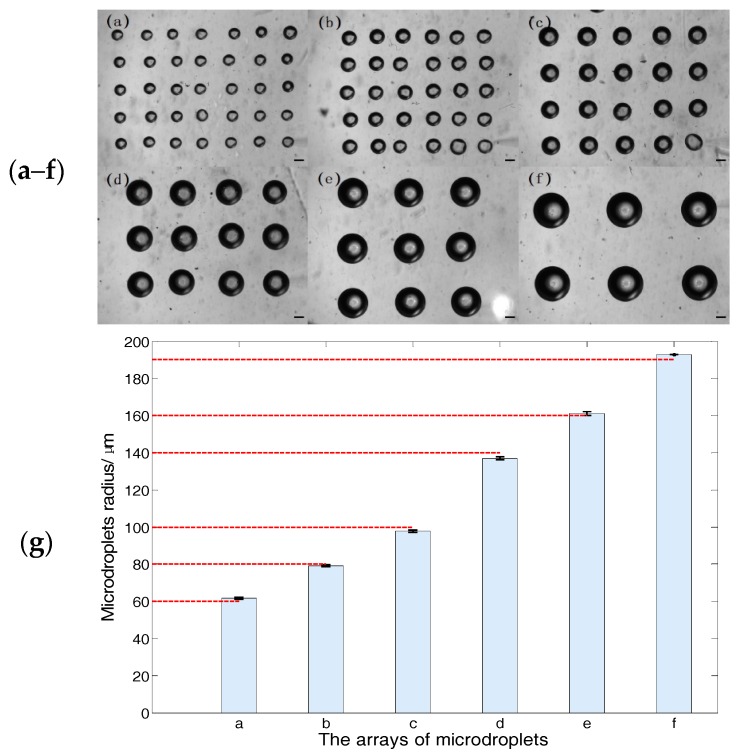
The experimental results and analysis. (**a**–**f**) The bright-field image of microdroplets, the scale bar is 100 μm (see Supplementary Video S1); (**g**) the uniformity analysis of the microdroplets’ radii, the column denotes the radius mean and the dashed red lines are the desired radii. The error bars are standard errors, which are 0.83%, 0.56%, 0.7%, 0.64%, 0.63%, and 0, respectively.

In the first experiment, the radius at the micropipette opening is 2.2 μm, and the desired microdroplet radii are 60 μm, 80 μm, 100 μm, 140 μm, 160 μm, and 190 μm, respectively. Thus, the injection times are 0.9 s, 1.9 s, 3.5 s, 9 s, 13 s, and 21.5 s, respectively, which are calculated according to Equation (11), with the given injection pressure of 9 kPa. The six arrays of microdroplets are shown as Figure 11a–f and the uniformity analysis of the microdroplet radii is shown as Figure 11g. The mean radius of six arrays of microdroplets are 62 μm, 79 μm, 98 μm, 134 μm, 161 μm, and 193 μm, respectively, and the standard error of the six arrays of microdroplets are 0.83%, 0.56%, 0.7%, 0.64%, 0.63%, and 0, respectively.

**Figure 12:**
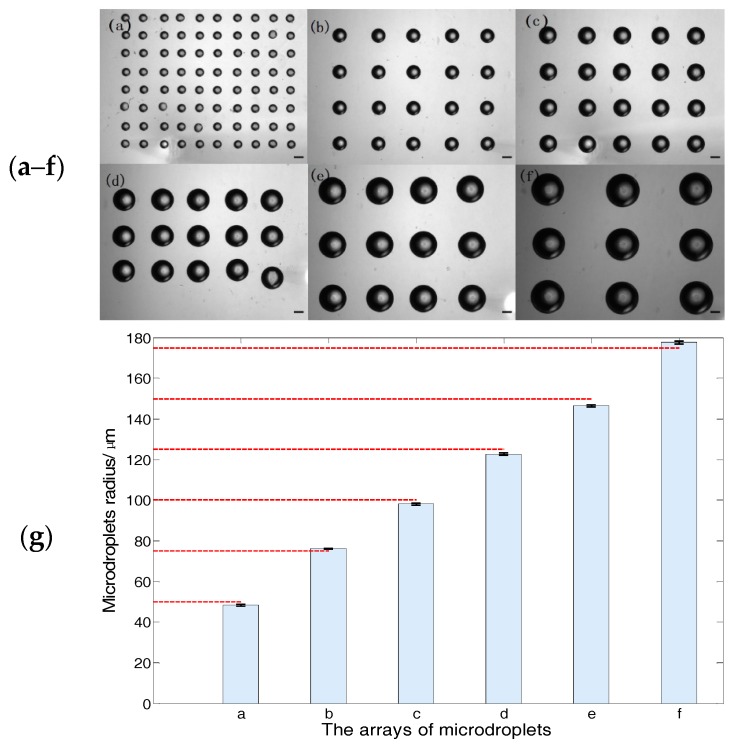
The experimental results and analysis. (**a**–**f**) The bright-field image of microdroplets, the scale bar is 100 μm (see Supplementary Video S2). (**g**) the uniformity analysis of the microdroplets’ radii, the column denotes the radius mean and the dashed red lines are the desired radii. The error bar are the standard errors, which are 0.66%, 0.33%, 0.38%, 0.46%, 0.33%, and 0.43%, respectively.

In the second experiment, the radius at the micropipette opening is 3.5 μm, and the desired microdroplet radii are 50 μm, 75 μm, 100 μm, 125 μm, 150 μm, and 175 μm, respectively. Thus, the injection pressure is 0.31 kPa, 0.9 kPa, 1.92 kPa, 3.45 kPa, 5.57 kPa, and 8.35 kPa, respectively, which are calculated according to Equation (11), with the given injection time of 6 s. The six arrays of microdroplets are shown as Figure 12a–f and the uniformity analysis of the microdroplet radii is shown as Figure 12g. The mean radius of six arrays of microdroplets are 48 μm, 76 μm, 98 μm, 123 μm, 146 μm, and 178 μm, respectively, and the standard error of six arrays of microdroplets are 0.66%, 0.33%, 0.38%, 0.46%, 0.33%, and 0.43%, respectively.

In addition, the experiment results show that the microdroplets are fixed on the substrate in the form of spheres, which verified the hybrid substrate, according to Figure 11 and Figure 12.

The standard errors in the two experiments are all less than 2% and the errors between the mean and desired microdroplet radii all fall in the range of ±5%. Therefore, these experimental results demonstrate that the proposed relationship and substrate-contacting method are applicable for size-adjustable microdroplet generation.

## 4. Conclusions

We demonstrated a size-adjustable microdroplet generation method successfully. In this method, a CI model is proposed and used to quantify the critical pressure and improve the effectiveness of this method. We carry out five groups of experiments based on the substrate-contacting method with one micropipette, with 1600 images captured within each group and the microdroplet radius of each image is detected. Then, we determined the relationship among the microdroplet radius, the radius at the micropipette opening, time, and pressure from the experimental results. To verify the relationship, we conducted the two more experiments; the excellent quality of the fit for the two experimental results verify the universality of the relationship.

The relationship is the critical step for size-adjustability of microdroplet generation. To verify the effectiveness of the relationship, we conducted two experiments; each of the experiments has six desired microdroplet radii. In the one experiment, we adjust the injection time with a given injection pressure, the desired microdroplet radii are 60 μm, 80 μm, 100 μm, 140 μm, 160 μm, and 190 μm. Correspondingly, the mean radii of the microdroplets are 62 μm, 79 μm, 98 μm, 137 μm, 161 μm, and 193 μm, respectively. The standard error of the six arrays of microdroplets are 0.83%, 0.56%, 0.7%, 0.64%, 0.63%, and 0, respectively. In the other experiment, we adjusted the injection pressure with a given injection time, and the desired microdroplets’ radii are 50 μm, 75 μm, 100 μm, 125 μm, 150 μm, and 175 μm. Correspondingly, the mean radii of the microdroplets are 48 μm, 76 μm, 98 μm, 123 μm, 146 μm, and 178 μm, respectively. The standard error of the six arrays of microdroplets are 0.66%, 0.33%, 0.38%, 0.46%, 0.33%, and 0.43%, respectively.

The standard errors of the microdroplet radii are all less than 2% and the experimental errors between the mean and desired microdroplet radii fall in the range of ±3%, the average operating speed is 20 microdroplets/min and the minimum radius of the microdroplets is 25 μm.

Interestingly, the exponents in all equations are similar, approximately equal to one-third, with an excellent quality of fit. Thus, we think the injection pressure *P* is proportional to the flow rate: *P* is proportional to *R*^3^, with the shape of microdroplets being almost spherical, which is shown in Figure 4e. The relationship among *R*, *T*, *P*, and *r* is determined with *R =* 14.18*rP*^(1/3)^*T*^(1/3)^, according the 1/3 law. Using this formula, we predict the microdroplets radius in the size-adjustable microdroplet generation. The radius error scope falls in the range 10%–20%. The failure to predict the relationship among *R*, *T*, *P*, and *r* from 1/3 law implies that the microdroplets’ shapes are not perfectly spherical, as shown in Figure 4e, possibly due to the physical characteristics of the substrate, the oil density, and water solution density. This will be explored systematically in the future.

With no need for a heat source and external liquid pressure device, the method does not impact the nature of the experimental solution, which enhances the variety of injection liquids. This method is conducted only in an ordinary micro-operation platform without any complex additional apparatus. Overall, this new method enhances the flexibility in the adjustment of microdroplet size, and reduces the cost and operative difficulty.

## Figures and Tables

**Table 1:** The measured critical injection (CI) pressure versus *r*, and the theoretical CI pressure versus *r*, according to Equation (3).

*r* (μm)	Measured CI Pressure (kPa)	Theory CI Pressure (kPa)
2.08	15.63	13.52
2.42	10.50	11.62
2.77	12.63	11.15
3.11	8.21	9.04
3.46	7.72	8.13
4.50	5.60	6.25
5.19	5.30	5.42
5.53	5.17	5.09
7.27	3.66	3.87
11.76	2.41	2.39
16.26	1.62	1.73

**Table 2:** The fitting results of five experiments, with the radius at the micropipette opening of 2.2 μm. *T* (unit: s) is the injection time; *R* (unit: μm) is the radius of the microdroplet.

*P* (kPa)	Fitting Formula	Goodness of Fit	Fitting Formula	Goodness of Fit
6.73	$R=49.43T^{0.3604}$	0.9958	$R=49.3T^{0.36}$	0.991
8.21	$R=54.25T^{0.3596}$	0.9905	$R=53.48T^{0.36}$	0.989
9.7	$R=57.03T^{0.3560}$	0.9896	$R=55.74T^{0.36}$	0.9807
11.18	$R=60.12T^{0.3685}$	0.9883	$R=59.52T^{0.36}$	0.9805
12.67	$R=63.81T^{0.3576}$	0.9946	$R=62.98T^{0.36}$	0.9921

**Table 3:** The fitting results of five microdroplets. The radius at the micropipette opening is 3.5 μm. *T* (unit: s) is the injection time; *R* (unit: μm) is the radius of the microdroplet.

*P* (kPa)	Fitting Formula	Goodness of Fit
3.46	$R=64.15T^{0.36}$	0.99
4.65	$R=73.68T^{0.36}$	0.99
5.83	$R=80.67T^{0.36}$	0.99
7.03	$R=86.03T^{0.36}$	0.99
8.21	$R=91.58T^{0.36}$	0.99

**Table 4:** The fitting results of five microdroplets. The radius at the micropipette opening is 6.9 μm. *T* (unit: s) is the injection time; *R* (unit: μm) is the radius of the microdroplet.

*P* (kPa)	Fitting Formula	Goodness of Fit
1.68	$R=100.8T^{0.36}$	0.99
2.57	$R=117.1T^{0.36}$	0.99
3.46	$R=129.5T^{0.36}$	0.99
4.35	$R=141.5T^{0.36}$	0.99
5.24	$R=152.8T^{0.36}$	0.99

## Supplementary Materials

The following are available online at www.mdpi.com/2072-666X/8/3/88/s1, Video S1: The process of microdroplets generation for Figure 11a–f; Video S2: The process of microdroplets generation for Figure 12a–f.

## References

[1] Theberge A.B., Courtois F., Schaerli Y., Fischlechner M., Abell C., Hollfelder F., Huck W.T. (2010). Microdroplets in microfluidics: An evolving platform for discoveries in chemistry and biology. Angew. Chem. Int. Ed..

[2] Maeda K., Onoe H., Takinoue M., Takeuchi S. (2012). Controlled synthesis of 3D multi-compartmental particles with centrifuge-based microdroplet formation from a multi-barrelled capillary. Adv. Mater..

[3] Ng K.C., Whitten W.B., Arnold S., Ramsey J.M. (1992). Digital chemical analysis of dilute microdroplets. Anal. Chem..

[4] Maeki M., Yamaguchi H., Yamashita K., Nakamura H., Miyazaki M., Maeda H. (2011). Analysis of kinetic behavior of protein crystallization in nanodroplets. Chem. Lett..

[5] Um E., Lee S.G., Park J.K. (2010). Random breakup of microdroplets for single-cell encapsulation. Appl. Phys. Lett..

[6] Weitz M., Mückl A., Kapsner K., Berg R., Meyer A., Simmel F.C. (2014). Communication and computation by bacteria compartmentalized within microemulsion droplets. J. Am. Chem. Soc..

[7] Garstecki P., Fuerstman M.J., Stone H.A., Whitesides G.M. (2006). Formation of droplets and bubbles in a microfluidic T-junction-scaling and mechanism of break-up. Lab Chip.

[8] Xu J.H., Li S.W., Tan J., Wang Y.J., Luo G.S. (2006). Preparation of highly monodisperse droplet in a T-Junction microfluidic device. AIChE J..

[9] Wang K., Lu Y.C., Xu J.H., Tan J., Luo G.S. (2010). Generation of micromonodispersed droplets and bubbles in the capillary embedded T-junction microfluidic devices. AIChE J..

[10] Hsiung S.K., Chen C.T., Lee G.B. (2006). Micro-droplet formation utilizing microfluidic flow focusing and controllable moving-wall chopping techniques. J. Micromech. Microeng..

[11] Wu N., Zhu Y., Leech P.W., Sexton B.A., Brown S., Easton C. (2007). Effects of surfactants on the formation of microdroplets in the flow focusing microfluidic device. Proc. SPIE Int. Soc. Opt. Eng..

[12] Jiang C.Y., Dong L., Zhao J.K., Hu X., Shen C., Qiao Y., Zhang X., Wang Y., Ismagilov R.F., Liu S.J. (2016). High-throughput single-cell cultivation on microfluidic streak plates. Appl. Environ. Microbiol..

[13] Barbulovic-Nad I., Lucente M., Sun Y., Zhang M., Wheeler A.R., Bussmann M. (2006). Bio-microarray fabrication techniques—A review. Crit. Rev. Biotechnol..

